# Temporal Heterogeneity of HER2 Expression and Spatial Heterogeneity of ^18^F-FDG Uptake Predicts Treatment Outcome of Pyrotinib in Patients with HER2-Positive Metastatic Breast Cancer

**DOI:** 10.3390/cancers14163973

**Published:** 2022-08-17

**Authors:** Chengcheng Gong, Cheng Liu, Zhonghua Tao, Jian Zhang, Leiping Wang, Jun Cao, Yannan Zhao, Yizhao Xie, Xichun Hu, Zhongyi Yang, Biyun Wang

**Affiliations:** 1 Department of Breast and Urological Medical Oncology, Fudan University Shanghai Cancer Center, Shanghai 200032, China; 2 Department of Oncology, Shanghai Medical College, Fudan University, Shanghai 200032, China; 3 Department of Nuclear Medicine, Fudan University Shanghai Cancer Center, Shanghai 200032, China; 4 Center for Biomedical Imaging, Fudan University, Shanghai 200032, China; 5 Shanghai Engineering Research Center of Molecular Imaging Probes, Shanghai 200032, China

**Keywords:** metastatic breast cancer, heterogeneity, HER2, ^18^F-fluorodeoxyglucose positron emission tomography/computed tomography, pyrotinib, therapy response

## Abstract

**Simple Summary:**

Tumor heterogeneity plays an important role in malignant behaviors and treatment responses. This study aimed to evaluate the temporal and spatial heterogeneity in clinical practice and investigate its impact on the treatment outcome of pyrotinib in patients with HER2-positive metastatic breast cancer. Temporal heterogeneity was evaluated by the discordance between primary and metastatic immunohistochemistry results. ^18^F-FDG uptake heterogeneity on baseline PET/CT scan was assessed to reflect spatial tumor heterogeneity among metastases. Our results showed that heterogeneous HER2 status between primary and metastatic lesions and spatial ^18^F-FDG uptake heterogeneity were predictive of poorer outcomes of pyrotinib treatment. The best method to evaluate tumor heterogeneity in clinical practice still needs to be identified. Temporal heterogeneity of HER2 expression and spatial heterogeneity of ^18^F-FDG uptake provided practically applicable methods to assess tumor heterogeneity and potential guidance for treatment decisions.

**Abstract:**

Background: This study aimed to evaluate tumor heterogeneity of metastatic breast cancer (MBC) and investigate its impact on the efficacy of pyrotinib in patients with HER2-positive MBC. Methods: MBC patients who underwent ^18^F-FDG PET/CT before pyrotinib treatment were included. Temporal and spatial tumor heterogeneity was evaluated by the discordance between primary and metastatic immunohistochemistry (IHC) results and baseline ^18^F-FDG uptake heterogeneity (intertumoral and intratumoral heterogeneity indexes: HI-inter and HI-intra), respectively. Progression-free survival (PFS) was estimated by the Kaplan–Meier method and compared by a log-rank test. Results: A total of 572 patients were screened and 51 patients were included. In 36 patients with matched IHC results, 25% of them had HER2 status conversion. Patients with homogenous HER2 positivity had the longest PFS, followed by patients with gained HER2 positivity, while patients with HER2 negative conversion could not benefit from pyrotinib (16.8 vs. 13.7 vs. 3.6 months, *p* < 0.0001). In terms of spatial heterogeneity, patients with high HI-intra and HI-inter had significantly worse PFS compared to those with low heterogeneity (10.6 vs. 25.3 months, *p* = 0.023; 11.2 vs. 25.3 months, *p* = 0.040). Conclusions: Temporal heterogeneity of HER2 status and spatial heterogeneity of ^18^F-FDG uptake could predict the treatment outcome of pyrotinib in patients with HER2-positive MBC, which provide practically applicable methods to assess tumor heterogeneity and guidance for treatment decisions.

## 1. Introduction

Breast cancer (BC) remains the most common cancer and the leading cause of cancer-related death in women worldwide. Approximately, 15–20% of BCs are human epidermal growth factor receptor 2 (HER2)-positive, which used to be considered an aggressive phenotype with poor prognosis until the development of anti-HER2 targeted therapy [1,2,3,4,5].

Pyrotinib is an orally available, irreversible pan-Erb receptor tyrosine kinase inhibitor that targets HER1, HER2, and HER4. The phase II study demonstrated that the combination of pyrotinib and capecitabine significantly prolonged the PFS of patients with HER2-positive MBC previously treated with taxanes, anthracyclines, and/or trastuzumab compared with lapatinib and capecitabine (18.1 months vs. 7.0 months, hazard ratio, 0.36; 95% confidence interval [CI], 0.23–0.58; *p* < 0.001) [6]. Based on the impressive improvement in PFS, pyrotinib has been granted accelerated but conditional approval for the treatment of metastatic HER2-positive BC, regardless of prior exposure to trastuzumab, in China in August 2018. PHENIX, a double-blinded, multicenter, randomized phase III study, showed that pyrotinib plus capecitabine significantly prolonged PFS (11.1 months vs. 4.1 months, *p* < 0.001) and had a better overall response rate (ORR) (68.6% vs. 16.0%, *p* < 0.001) than capecitabine monotherapy [7]. PHOBE, another phase III randomized controlled trial of pyrotinib, directly compared pyrotinib and capecitabine with lapatinib and capecitabine in HER2-positive MBC patients who had been previously treated with trastuzumab and taxanes. The median PFS of pyrotinib and capecitabine was 12.5 months, significantly longer than that of 6.8 months in lapatinib (*p* < 0.0001) [8]. Pyrotinib gained full approval in July 2020 based on the results of the PHENIX and PHOBE trials and has been covered by national medical insurance since November 2019. 

Due to the fact that trastuzumab emtansine (T-DM1) has not been approved in China for the treatment of MBC until June 2021 and is not covered by national medical insurance till now, pyrotinib has been an important treatment option for HER2-positive MBC. A multicenter, observational, large-scale, real-world study has been conducted to evaluate the efficacy of pyrotinib in China in daily clinical practice [9]. Among 862 MBC patients enrolled in this study, 31.1%, 35.7% and 33.2% received pyrotinib as first-line, second-line and third- or later-line treatment, respectively.

Despite the promising results in clinical trials, not all patients benefited from pyrotinib treatment in real-world clinical practice [10,11]. Therefore, it is important to identify biomarkers to predict response to pyrotinib-based therapy as it may lead to optimization of treatment selection strategy for thousands of patients in China.

Tumor heterogeneity plays an important role in the malignant behaviors and treatment responses of different cancers [12,13,14,15]. At an individual level, tumor heterogeneity can manifest as temporal heterogeneity, the molecular evolution of the tumor over time, and as spatial heterogeneity, which describes the uneven distribution of genetically diverse tumor subpopulations across different disease sites or within a single disease site or tumor [16].

Tumor heterogeneity can be detected by conventional immunohistochemistry (IHC), gene expression profiling, or other methods. In breast cancer, the discordance of estrogen receptor (ER), progesterone receptor (PR), and HER2 expression levels between matched primary and metastatic lesions could reflect temporal intratumor heterogeneity. A meta-analysis evaluated receptor discordance rates between primary and metastatic breast cancer in 47 studies with 3384 paired samples. The median discordance rates for ER, PR and HER2 were 14% (0–67%), 21% (0–62%) and 10% (0–44%), respectively [17]. A large-scale real-world study has compared matched IHC results in 1677 MBC patients and reported a change in HR and HER2 expression of 14.2% and 7.8%. In terms of subtypes, more than half of patients (53%) with primary HR+/HER2+ disease showed status change [18]. As a therapeutic target, the evaluation of HER2 is of great importance. Another meta-analysis evaluated the HER2 status in the primary tumor and corresponding distant metastasis in 35 studies. The discordance rate was assessed in 2440 patients for HER2. The studies were subdivided into three groups—studies using FISH only, studies using IHC only, and studies using a combination of IHC and FISH (in case of 2+/equivocal IHC)—to assess receptor status. No significant difference was seen between the total discordance percentages of these groups (*p* = 0.25) [19]. Despite technical reasons that may affect the examination of IHC results, the discordance of ER, PR and HER2 between primary and metastatic disease is considered a truly existing biological phenomenon. Tumor heterogeneity is one of the most important reasons behind this phenomenon. Due to the fact that therapeutic strategy is highly dependent on the IHC evaluation of these markers, the re-biopsy of the metastatic lesion, especially when metastasis is diagnosed for the first time, has been recommended by several international guidelines.

In terms of spatial heterogeneity, however, multiple biopsies of different metastatic sites or multi-region sampling within a single lesion are required for comprehensive assessment, which could not be widely adopted owing to prohibitive risks of biopsy. In this case, functional molecular imaging can serve as an alternative option to characterize tumor spatial heterogeneity noninvasively. ^18^F- fluorodeoxyglucose (FDG) PET-CT provides the metabolic activity of various lesions, which could reflect regional variation in tumor function in solid tumors. The predictive value of intratumoral heterogeneity of baseline ^18^F- FDG uptake in various tumors has been proved [20,21,22,23,24]. Common methods to examine this include textural analysis, coefficient of variance (COV), cumulative standardized uptake value (SUV)-volume histogram (CSH), the area under the CSH, and fractal analysis [25,26,27,28,29,30]. However, these methods are still too complicated to be widely applicable for metastatic disease in clinical practice, particularly if there are multiple metastatic lesions. Our previous study introduced simplified quantitative parameters to represent the inter- and intratumoral heterogeneous characteristics of metastatic disease and proved their value in predicting the response to treatment in patients with triple-negative and hormone receptor (HR)-positive BC [31,32,33,34]. In this study, we evaluated ^18^F-FDG uptake heterogeneity in HER2 positive MBC to reflect spatial tumor metabolic heterogeneity among metastases and explored its predictive value for the treatment outcome of pyrotinib.

This study aimed to evaluate the temporal heterogeneity between primary and metastatic lesions and spatial heterogeneity among metastatic lesions in HER2-positive MBC and to explore their ability to predict patient outcomes under pyrotinib treatment.

## 2. Materials and Methods

### 2.1. Patients

A total of 572 patients with MBC treated with pyrotinib in the Fudan University Shanghai Cancer Center (FUSCC) between 1 September 2018 and 24 July 2021 were screened. Patients who underwent whole-body FDG PET/CT within 4 weeks before the initiation of pyrotinib were included in this study. Patients without detailed medical history or who were lost to follow-up were excluded. Data were retrospectively obtained from the patients’ medical history. 

### 2.2. IHC Evaluation 

The ER, PR and HER2 status was derived from pathological reports. According to the Standard Operating Procedure (SOP) of FUSCC, pathology consultation should be recommended before initiating treatment for patients who were not diagnosed in our center, except for those who were not able to provide archived tumor tissue. Pathology reports were evaluated through an independent review of two committee-certified pathologists with expertise in breast cancer. The discrepancies between the two pathologists were resolved through a review of a third pathologist. Immunohistochemical staining for ER, PR, HER2 was performed with antibodies against ER (SP1, Roche Ventana), PR (IE2, Roche Ventana), HER2 (4B5, Roche Ventana), as previously reported [35]. PathVysion HER2 DNA Probe Kit (Abbott Molecular, Abbott Park, Illinois) was used for HER2 FISH following the manufacturer’s instructions [35]. HR positivity was defined according to national guidelines with a cutoff level of 1% [36]. HR status in this article was defined as “positive” or “negative”. For further exploration, tumors were stratified into four groups based on the percentage of ER+: ER negative (<1%), low ER (1–10%), intermediate ER (10–50%) and high ER (>50%). “HR expression change” in this article refers to the change between low, intermediate and high expression of HR. HER2 status was interpreted using the updated 2018 ASCO/CAP guideline recommendations for HER2 testing, based on IHC and FISH results [37]. For further exploration, tumors were stratified into four groups based on HER2 IHC results: HER2 negative (0), HER2 low (+, ++ and FISH-), HER2 positive with IHC (+~++, FISH+) and HER2 positive with IHC (+++). “HER2 expression change” in this article refers specifically to the conversion between these groups. The subtype referred to in this study included HR+/HER2+, HR-/HER2+, HR+/HER2− and HR-/HER2− based on the defined thresholds.

### 2.3. PET/CT Imaging

^18^F-FDG was produced automatically by cyclotron (Siemens CTI RDS Eclips ST, Siemens, Knoxville, TN, USA) using the Explora FDG4 module in our center. The radiochemical purity was over 95%.

Patients were required to fast for at least 6 h before the ^18^F-FDG PET/CT scan, and blood glucose levels were to be <200 mg/dL at the time of injection. Sixty minutes following intravenous ^18^F-FDG administration (mean dose 3.7–7.4 MBq/kg), patients underwent PET/CT from the mid-skull to the mid-thigh (Siemens Biograph 16HR PET/CT or mCT Flow PET/CT scanner, Siemens Medical solutions, USA). Low-dose CT was performed during tidal breathing to correct for attenuation, followed by a PET emission scan that covered the identical transverse field of view.

### 2.4. Image Interpretation

^18^F-FDG PET/CT images were reviewed and evaluated independently by two board-certified nuclear medicine physicians using a multimodality computer platform (Syngo, Siemens, Knoxville, TN, USA). In the event of disagreement between the two readers, a consensus was reached on a final reading for the statistical analyses. All hypermetabolic metastatic lesions were picked for analysis, whereas hypermetabolic foci judged to be inflammation or normal physiological activity were not considered.

Semiquantitative analysis of tumor metabolic activity was obtained using SUV normalized to body weight. The maximum SUV (SUVmax) and mean SUV (SUVmean) for each metastatic lesion were recorded by manually placing an individual region of interest (ROI) around each tumor on all consecutive slices that contained the lesion on co-registered and fused transaxial PET/CT images. The SUVmax across all metastatic lesions was then evaluated. Then, the metabolic tumor volume (MTV) was automatically extracted from the software based on an SUV threshold of 40. Total lesion glucose (TLG) was calculated according to the formula: TLG = SUVmean × MTV. A quantitative measure of intratumoral heterogeneity, the intratumoral heterogeneity index (HI-intra), was measured by dividing the SUVmax of each lesion by the SUVmean of that lesion [31,34,38,39]. The mean HI-intra of all lesions was selected to represent the intratumoral heterogeneity for each patient. Intertumoral heterogeneity was evaluated by the COV and intertumoral heterogeneity index (HI-inter), another parameter we proposed. The COV of metastatic lesions was calculated from the SUVmax of every ROI as the ratio of the standard deviation to the mean [40]. The HI-inter was the maximum value of the SUVmax divided by the minimum value of the SUVmax for all metastatic lesions [32]. Considering the partial volume effect and repeatability, only lesions no less than 10 mm in diameter were included in further analysis. Bone lesions with confirmation by CT or magnetic resonance imaging were included.

### 2.5. Statistical Analyses

Data are presented as medians (ranges) or numbers of patients (percentages). Treatment outcome was assessed as PFS, which was measured from the date of pyrotinib initiation to the first documented disease progression or death. Disease progression was determined by the Response Evaluation Criteria in Solid Tumors version 1.1. Overall survival (OS) was measured from the date of pyrotinib initiation to the date of death or the last follow-up. Kaplan–Meier method was conducted for estimating survivals and log-rank test for comparisons. Mann–Whitney U test was applied for comparison between groups with quantitative variables with non-normal distribution. Analyses of factors potentially associated with temporal and spatial tumor heterogeneity were performed using the Chi-squared test or the Fisher exact test. 

Time-dependent survival receiver operating characteristic (ROC) analysis had an advantage in assessing the prognostic value of the biomarkers and determining optimal cutoff values by maximizing both sensitivity and specificity of the event-time outcome [41]. PET/CT parameters cutoff values were determined by survival ROC library in R. Other statistical analyses were conducted by SPSS IBM^®^ version 22 (SPSS Inc., Chicago, IL, USA). All *p*-values were two-sided, and *p* < 0.05 was considered significant.

## 3. Results

### 3.1. Patient Baseline Characteristics and Their Association with PFS

There were 51 MBC patients that met the criteria of undertaking ^18^F-FDG PET/CT within 4 weeks before the initiation of pyrotinib and were included in the analysis. The demographic and clinical characteristics of the patients are summarized in Table 1.

The median patient age was 54 years (range 23–74 years). 39.2% of the patients had HR-positive disease. Six patients were diagnosed with de novo stage IV disease. In patients who received radical treatment, 68.9% had disease relapse within two years. Twenty-one patients (41.2%) had ≥3 metastatic sites, and the common sites of metastases were the bone (45.1%), liver (23.5%), brain (19.6%) and lung (19.6%). Around half of the patients (49.0%) had visceral involvement. Most patients received pyrotinib as the first or second treatment (86.3%). Of the patients, 78.4% received pyrotinib and capecitabine and other patients received combinational agents such as vinorelbine. Additionally, 92.2% of the patients had been treated with trastuzumab and 25.5% of the patients had prior pertuzumab exposure. 

At the time of analysis, 26 patients had documented disease progression (51%). The median PFS was 13.7 months (95% CI, 9.3–18.2). The data for OS were immature at the time of analysis. In 41 patients with evaluable disease, the objective response rate was 48.8%.

The associations between clinical factors and PFS are shown in Table 2. Patients who received pyrotinib as first or second-line treatment had a significantly longer median PFS than patients who received pyrotinib as third- or later-line treatment (15.7 vs. 10.6 months, *p* = 0.017). Patients with one metastatic site had better outcomes compared with patients with a higher tumor burden (25.3 vs. 11.2 months, *p* = 0.015). HR status did not affect the PFS of pyrotinib treatment (13.7 vs. 13.4 months, *p* = 0.930). Tumors were stratified into four groups based on the percentage of ER+ percentage on the most recent IHC results: ER negative (<1%, *n* = 33), low ER (1–10%, *n* = 4), intermediate ER (10–50%, *n* = 5) and high ER (>50%, *n* = 9). The median PFS for these patients were 13.4 months, 10.2 months, 16.8 months and 15.7 months, respectively (*p* = 0.343). It seems that patients with low ER had the worse outcome. Tumors were stratified into four groups based on HER2 expression on the most recent IHC results: HER2 negative (0, *n* = 1), HER2 low (+, ++ and FISH-, *n* = 3), HER2 positive with IHC (+~++, FISH+) (*n* = 12), HER2 positive with IHC (+++) (*n* = 35). The median PFS for these patients were 3.6 months, 5.8 months, 13.7 months and 16.8 months, respectively (*p* < 0.0001).

### 3.2. Temporal Tumor Heterogeneity and Its Association with PFS

Of patients enrolled in this study, 88.2% (45/51) had tumor IHC results confirmed by the Department of Pathology in FUSCC. Among 51 patients enrolled in this study, 46 of them (90.2%) had had re-biopsy before the initiation of pyrotinib and 36 patients (70.6%) had matched primary and metastatic IHC results. Thus, the following evaluation of temporal heterogeneity in terms of IHC was performed in 36 patients with matched IHC results. There were 24 patients’ primary sites and metastases IHC results that had both been evaluated in FUSUCC.

The discordance rate for HR and/or HER2 status between primary and metastases was 41.7% (15/36).

**Figure 1:**
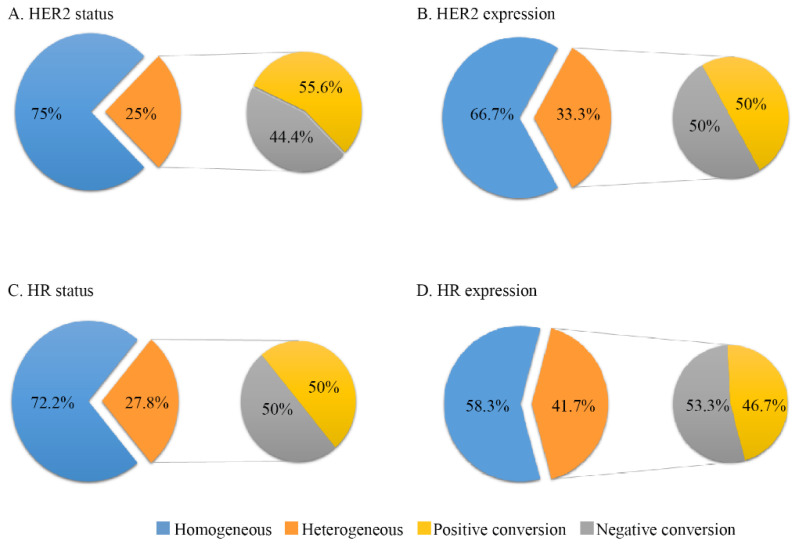
Temporal tumor heterogeneity in terms of IHC discordance between primary and metastatic tumors. (**A**) HER2 status. (**B**) HER2 expression. (**C**) HR status. (**D**) HR expression. Abbreviations: IHC, immunohistochemistry; HR, hormone receptor expression; HER2, human epidermal growth factor receptor 2. HR expression change was defined as conversion between HR negative (<1%), low HR (1–10%), intermediate HR (10–50%) and high HR (>50%). HER2 expression change was defined as conversion between HER2 negative (0), HER2 low (+, ++ and FISH−), HER2 positive with IHC (+~++, FISH+) and HER2 positive with IHC (+++).

The change rate of HER2 status was 25% (9/36), with a positive conversion of 55.6% (5/9) and a negative conversion of 44.4% (Figure 1). Twelve patients (33.3%) showed heterogeneous HER2 expression between primary and metastatic IHC, with a change of gain in 50% and loss in 50%. The change rate for HR status was 27.8% (10/36), with a positive conversion and a negative conversion of 50% each. Fifteen patients (41.7%) showed heterogenous HR expression between primary and metastatic IHC, with a gain of 46.7% and a loss of 53.3%.

In terms of subtype, 41.7% of patients (15/36) showed disordinate subtypes between primary and metastatic lesions. In 21 patients with HR-/HER2+ primary disease, 15 patients (71.4%) showed homogenous IHC results in metastatic sites, while 6 patients changed into HR+/HER2+ (*n* = 2), HR+/HER2− (*n* = 2), and HR−/HER2− (*n* = 2). In 10 patients with HR+/HER2+ breast cancer, 6 patients (60%) remained HR+/HER2+, while 4 patients had HR loss and changed into HR−/HER2+ disease. In addition, 3 of 4 patients with HR+/HER2− primary disease changed into HR+/HER2+ subtype and one patient changed into HR−/HER2+ subtype. One patient with HR−/HER2− breast cancer changed into HR+/HER2+ subtype in metastatic disease.

The association between baseline clinical factors and temporal heterogeneity in terms of HER2 status was evaluated and shown in Table S1. HER2 status discordance was not associated with the treatment line or resistance to previous trastuzumab.

**Figure 2:**
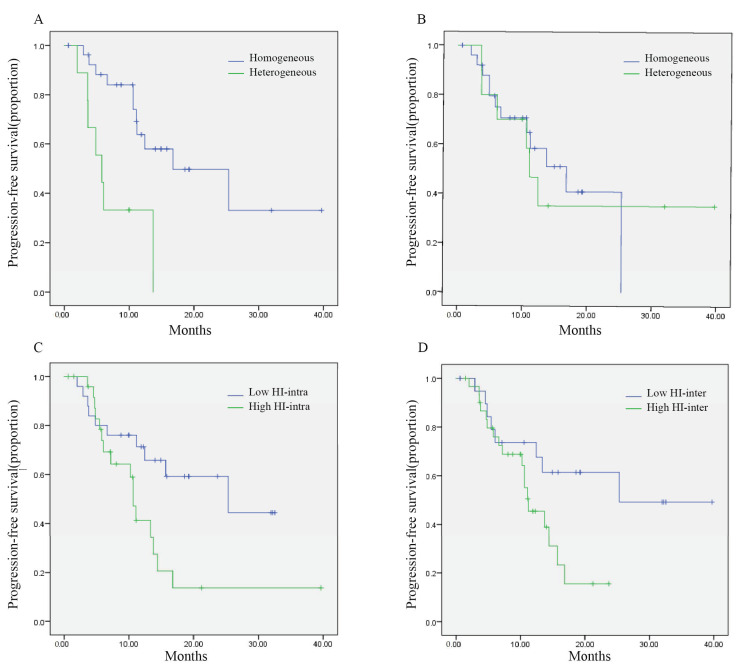
Kaplan–Meier curve of PFS stratified according to the temporal heterogeneity of IHC results (**A**,**B**) and spatial heterogeneity of ^18^F-FDG uptake (**C**,**D**) in patients with HER2-positive metastatic breast cancer. (**A**) HER2 status heterogeneity between primary and metastatic tumors. (**B**) HR status heterogeneity between primary and metastatic tumors. (**C**) Intratumoral heterogeneity of ^18^F-FDG uptake. (**D**) Intertumoral heterogeneity of ^18^F-FDG uptake. Abbreviations: PFS, progression-free survival; FDG, fluorodeoxyglucose; IHC, immunohistochemistry; FDG, fluorodeoxyglucose; HER2, human epidermal growth factor receptor 2; HR, hormone receptor; HI-intra, intratumoral heterogeneity index; HI-inter, intertumoral heterogeneity index.

Heterogeneity in HER2 status was significantly associated with shorter PFS of pyrotinib (5.8 vs. 16.8 months, *p* = 0.001, Figure 2A). Patients with HER2 negative conversion, positive conversion and homogenous status between primary and metastatic disease showed a median PFS of 3.6 months, 13.7 months and 16.8 months (*p* < 0.0001). Patients with discordant HER2 expression in IHC also showed worse outcomes (5.8 vs. NR, *p* = 0.001). The PFS for patients with HER2 loss, gain and unchanged were 4.8 months, 13.7 months and not reached, respectively (*p* < 0.0001).

Heterogeneity in HR status did not seem to affect the treatment outcome of pyrotinib-based treatment (11.1 vs. 16.8 months, *p* = 0.887, Figure 2B). Patients with HR negative conversion, positive conversion and homogenous status between primary and metastatic disease showed a median PFS of 11.1 months, 12.4 months and 16.8 months (*p* = 0.800). The discordance of HR expression was not associated with efficacy either (*p* = 0.541). The median PFS for patients with HR loss, gain and unchanged were 13.7 months, 12.4 and 16.8 months, respectively (*p* = 0.763).

Patients with phenotypic heterogeneity between primary and metastatic disease had worse PFS, though not significantly (11.1 vs. 25.3 months, *p* = 0.195). The median PFS for patients with metastatic HR+/HER2+, HR−/HER2+, HR+/HER2− and HR−/HER2− disease was 16.8 months, 11.2 months, 3.6 months and 2.0 months, respectively (*p* = 0.0003).

Seven patients had multiple IHC results of the breast prior and after neoadjuvant therapy. The associations between HR, HER2 conversion and PFS of pyrotinib were consistent if changes during neoadjuvant therapy were also included in temporal tumor heterogeneity analyses.

### 3.3. Spatial Tumor Heterogeneity and Its Association with PFS

Comprehensive assessment of spatial intratumor heterogeneity required multiple biopsies of different metastatic sites or multi-region sampling within a single lesion, which was difficult to obtain due to practical reasons. Among patients enrolled in this study, only eight patients were able to evaluate spatial tumor heterogeneity in terms of IHC. 

Functional molecular imaging offers an alternative option to characterize tumor spatial heterogeneity in a noninvasive way. ^18^F- FDG PET/CT could demonstrate the metabolic activity of various metastatic lesions at once. A total of 318 metastatic lesions on baseline PET-CT were measured and analyzed. The optimal cutoff values of PET/CT parameters were determined by time-dependent survival ROC analysis. 

The association between baseline clinical factors and spatial heterogeneity in terms of FDG uptake was shown in Table S1. HI-intra was not associated with any bassline tumor characteristics. Patients with high HI-inter were more common in those with multiple metastases (≥2) and visceral metastasis. 

**Figure 3:**
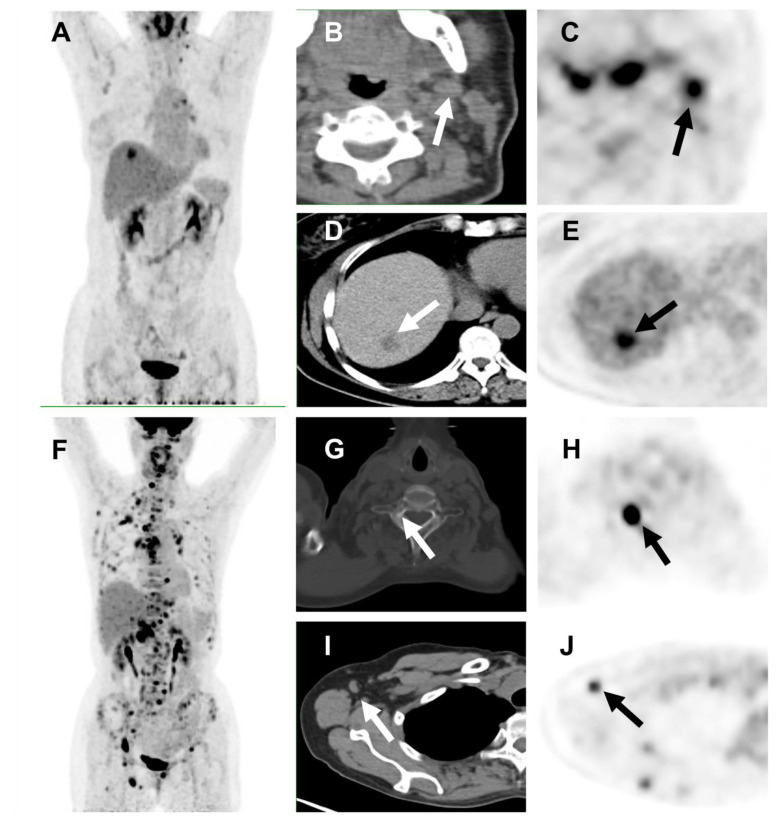
Representative cases of tumoral heterogeneity on ^18^F-FDG PET/CT and response to pyrotinib. (**A**–**E**) A 56-year-old woman underwent ^18^F-FDG PET/CT ((**A**), maximum intensity projection [MIP] image). The left cervical lymph node lesion had the highest uptake (**B**), CT image; (**C**), PET image; SUVmax = 7.94, SUVmean = 5.09), whereas the liver lesion had the lowest uptake ((**D**), CT; (**E**), PET, SUVmax = 6.94, SUVmean = 3.84). This patient’s median HI-intra was 1.68, and her HI-inter was 1.14. She has received pyrotinib treatment for 39.7 months and has not yet experienced tumor progression. (**F**–**J**) A 61-year-old woman who underwent ^18^F-FDG PET/CT ((**F**), MIP) showed multiple bone and lymph nodes metastases, with the highest uptake in the cervical vertebrae ((**G**), CT; (**H**), PET image; SUVmax = 21.72, SUVmean = 13.16) and the lowest uptake in the left axillary lymph node ((**I**), CT; (**J**), PET; SUVmax = 4.73, SUVmean = 3.19). This patient’s median HI-intra was 1.70, and her HI-inter was 4.59. She experienced disease progression after 3.6 months of pyrotinib treatment. Abbreviations: FDG, fluorodeoxyglucose; PET/CT, positron emission tomography/computed tomography; SUVmax, maximum standardized uptake value; SUVmean, mean standardized uptake value; HI-intra, intratumoral heterogeneity index; HI-inter, intertumoral heterogeneity index.

Patients with a high HI-intra (>1.69) had a median PFS of 10.6 months, which was significantly shorter than patients with low HI-intra (PFS: 25.3 months, *p* = 0.023, Figure 2C). Univariate analysis showed that patients with higher intertumoral heterogeneity (measured by classical COV) had worse PFS (11.1 months vs. 25.3 months, *p* = 0.026, Table S2). Simplified measurement of intertumoral heterogeneity- HI -inter could also discriminate patients into two groups (11.2 months and 25.3 months, *p* = 0.040, Figure 2D). Representative examples of patients’ images are shown in Figure 3.

Exploratory analysis to investigate the predictive value of other PET parameters was conducted and shown in Table S2. SUVmax uptake and TLG were also significantly associated with PFS. The median PFS of patients with high SUVmax (>7.96) was significantly shorter than that of patients with low SUVmax (11.1 months vs. Not reached, *p* = 0.008). Higher TLG was also associated with significantly shorter PFS (11.2 months vs. Not reached, *p* = 0.024). SUVmean and MTV, on the other hand, were not predictive for PFS.

### 3.4. Association between Temporal and Spatial Heterogeneity

The association between temporal and spatial tumor heterogeneity was shown in Table S3. In terms of temporal tumor heterogeneity, patients with heterogeneous HER2 status between primary and metastatic disease showed higher HI-inter value (z = −2.289; *p* = 0.022) and similar HI-intra value (z = −0.785, *p* = 0.432).

In terms of spatial tumor heterogeneity, 36.4% of patients with high HI-inter showed temporal HER2 discordance compared to 7.1% of patients in the low HI-inter group (*p* = 0.062). In addition, all three patients with synchronous heterogenous IHC results were classified in the high HI-inter group.

Thirteen patients had received circulating tumor DNA (ctDNA) analysis and next generation sequencing (NGS) test before the treatment of pyrotinib and ten of them showed gene abnormalities, including TP53 mutation (*n* = 7), ERBB2 amplification (*n* = 6), PIK3CA mutations (*n* = 3), Myc amplification (*n* = 3) and BRCA2 mutation (*n* = 2). The proportion of abnormal NGS results in patients with high HI-inter and low HI-inter was 90% (9/10) and 33.3% (1/3), respectively (*p* = 0.108).

## 4. Discussion

Drug resistance has been a heated research topic for decades. Accumulating evidence suggests that tumor heterogeneity resulting from clonal evolution limits the efficacy of BC treatment [42,43,44,45]. Therefore, the assessment of tumor clonal heterogeneity could provide important information for the prediction of treatment outcomes. The best method to evaluate tumor heterogeneity in clinical practice still needs to be identified. HR and HER2 expression changes between primary and metastatic lesions may be the most evident demonstration of temporal tumor heterogeneity. Spatial tumor heterogeneity could be hard to evaluate due to the difficulty of multiple biopsies in practice. Functional molecular imaging offers an alternative and noninvasive method for characterizing tumor spatial heterogeneity. FDG uptake among metastatic lesions was under the influence of many factors, such as proliferation, vascularization, cellular hypoxia and necrosis. These factors are also fundamental physiological mechanisms of tumor behaviors and treatment resistance [46]. Thus, FDG uptake heterogeneity could reflect tumor biological heterogeneity to some extent. Our previous work introduced a simplified quantitative index, the HI, to represent the heterogeneous characteristics of metastatic disease and proved the predictive value of the baseline HI in patients with triple-negative breast cancer and HR+/HER2− MBC [32,33,34]. In this study, we first applied this method to patients with HER2-positive MBC.

HER2-positive BC is a highly heterogeneous disease. Pathologists have noticed cell-to-cell variations in HER2-positive tumors since HER2 was first introduced as a diagnostic marker. Over the years, guidelines of the American Society of Clinical Oncology/College of American Pathologists (ASCO/CAP) are continually making efforts to optimize the thresholds to define HER2 positivity [37]. Multiple studies have reported the intratumoral heterogeneity of HER2 gene amplification. One of the most crucial mechanisms of anti-HER2 treatment therapy resistance was the heterogeneous expression of the therapeutic target within the tumor. The clinical impact of the intratumoral heterogeneity of HER2 copy number levels and regional variation of HER2 gene amplification on the prognosis of patients and the efficacy of anti-HER2 targeted therapy has been studied [47,48,49,50]. Neoadjuvant treatment has provided an important platform for exploration. A phase II neoadjuvant trial of T-DM1 and pertuzumab conducted at the Dana-Farber Cancer Institute first defined HER2 heterogeneity as an area with ERBB2 amplification in >5% but <50% of tumor cells, or a HER2-negative area by FISH. Their results showed that none of the 10 patients with HER2 heterogeneity achieved a pathological complete response rate (pCR), whereas 55% of patients not classified as HER2 heterogeneous had a pCR (*p* < 0.0001) [51]. Biomarker analysis from the neoadjuvant KRISTINE study in HER2-positive early breast cancer also showed that pCRs were higher in patients with HER2 IHC (+++) disease than HER2 IHC (++) (60.8% vs. 20.0%). HER2 IHC 2+/3+ fraction, defined as the sum of IHC2+ and IHC3+ staining percentage, was also evaluated. Patients with homogeneous HER2 IHC 2+/3+ fraction (≥80%) had the highest pCR compared to those with focal (<30%) and variable fractions (30–79%) [52]. However, the intratumoral heterogeneity of HER2-positive MBC has not been fully examined.

In this study, we enrolled 51 patients treated with pyrotinib-based therapy with a whole-body PET/CT scan prior to treatment. The treatment lines of patients in this study (1st: 41.2%; 2nd: 45.1%; 3rd or later: 13.7%) were similar to those in the phase III trial of pyrotinib (1st: 43%, 2nd: 42%, 3rd: 16%), but earlier to those reported in real-world study (1st: 31.1%, 2nd: 35.7%, 3rd or later: 33.2%) [8,9]. A possible reason for the high rate of frontlines was selection bias. Only patients who underwent ^18^F-FDG PET/CT within 4 weeks before the initiation of pyrotinib were included in this study. Whole-body ^18^F-FDG PET/CT was more likely to be recommended for patients with suspicious metastatic disease, thus the proportion for patients who were first diagnosed was higher. The median PFS was 13.7 months (95% CI, 9.3–18.2) in this study, comparable to that reported in the phase III trial (12.5 months), further proving the efficacy of pyrotinib [9]. Vinorelbine has been an alternative combinational agent for pyrotinib in patients with previous exposure to capecitabine [53].

In this study, tumors were stratified into four groups based on HER2 IHC expression on the most recent IHC results: HER2 negative, HER2 low, HER2 positive with IHC (+~++) and HER2 positive with IHC (+++). The median PFS for these patients were 3.6 months, 5.8 months, 13.7 months and 16.8 months, respectively (*p* < 0.0001). In the MARIANNE study, MBC patients were randomized to first-line trastuzumab plus taxane, T-DM1 plus placebo, or T-DM1 plus pertuzumab. Biomarkers showed that focal HER2 expression (IHC 3+ or IHC 2+) was present in 3.8% of patients and was associated with numerically shorter PFS [54]. We also evaluated the association between ER expression and PFS of pyrotinib. Interestingly, we found that patients with ER low positivity (1–10%) had the shortest PFS, consistent with the previous report of neoadjuvant trastuzumab and chemotherapy [50].

Temporal tumor heterogeneity in this study was evaluated by the IHC conversion between primary and metastatic sites, the most common method used by clinicians in daily practice. In 36 patients with matched primary and metastatic IHC results, the change rate of HER2 status was 25%, with a positive conversion of 55.6% and a negative conversion of 44.4%. Heterogeneity in HER2 status was significantly associated with shorter PFS of pyrotinib (5.8 vs. 16.8 months, *p* = 0.001). Various discordant rates of HER2 status between primary and metastatic breast cancers have been reported [18,19]. Population selection strategy may affect this result since patients with HER2 negative conversion are less likely to be given anti-HER2 treatment in metastatic settings. The association between baseline clinical factors and HER2 status heterogeneity was evaluated (Table S1). HER2 status discordance was not associated with the treatment line or resistance to previous trastuzumab. Another study selected patients who were receiving trastuzumab and reported a HER2 status discordant rate of 37.8%, with 67.9% of patients gaining HER2 amplification and 32.1% losing HER2 expression. Patients with HER2 negative conversion had significantly lower PFS for taxane–trastumab–pertuzumab (PFS 5.5 months), compared to HER2 unchanged patients (PFS 9 months, *p* = 0.01) and patients with HER2 positive conversion (PFS 14 months, *p* = 0.01) [55]. However, patients with positive conversion (PFS =1.0 months) did not seem to benefit from later-line T-DM1 treatment (PFS for HER2 unchanged was 6.0 months, for HER2 negative conversion was 1.5 months). Our study showed that patients with homogenous HER2 positivity throughout the disease had the highest PFS of pyrotinib, followed by positive conversion and negative conversion (16.8 vs. 13.7 vs. 3.6 months, *p* < 0.0001). These studies showed that patients with HER2 gained amplification could benefit from trastuzumab/pertuzumab and pyrotinib treatment but might predict TDM1 resistance. However, both studies were retrospective studies with relatively small sample sizes. Caution should be taken when interpreting these results referring to the treatment outcome of HER2 gained amplifications. Regardless of HER2 status, our study also showed that heterogeneous HER2 expression level between primary and metastatic IHC was also associated with shorter PFS (5.8 vs. Not reached, *p* = 0.001). PFS for patients with HER2 IHC expression loss, gain and unchanged were 4.8 months, 13.7 months and not reached, respectively (*p* < 0.0001).

Spatial tumor heterogeneity was assessed by ^18^F-FDG uptake on PET/CT scan. Our results showed that baseline spatial heterogeneity could predict the treatment efficacy of pyrotinib in HER2-positive MBC. Patients with a high HI-intra had significantly shorter PFS than patients with a low HI-intra (10.6 months vs. 25.3 months; *p* = 0.023, Figure 1A). In terms of intertumoral heterogeneity, COV is the conventional method for discriminating heterogeneity, but it can be time-consuming to calculate with the presence of multiple metastases. Our results showed that our simplified method of HI-inter can also represent the intertumoral heterogeneity in patients with MBC. Univariate analysis showed that patients with a high HI-inter tended to have worse PFS than those with a low HI-inter (11.2 months and 25.3 months, *p* = 0.040). No significant association was found between HI-intra and baseline tumor characteristics. HI-inter, which reflects the heterogeneity among different metastatic lesions, was higher in patients with multiple metastases (≥2) and visceral metastasis (Table S1). Several studies have indicated that SUVmax was higher in visceral metastases but no correlation has been established between visceral metastasis and metabolic heterogeneity [56,57].

Our study demonstrated that tumor heterogeneity had a significant impact on the efficacy of pyrotinib, which was consistent with a previous finding from ctDNA analysis. Translational exploration of the phase I study of pyrotinib performed ctDNA analyses and target-capture deep sequencing in 37 patients with HER2-positive MBC treated with pyrotinib alone or in combination with capecitabine [58]. Patients with three or more mutation clusters (defined as high tumor heterogeneity in this article) had significantly worse PFS, with a median PFS of 30 weeks, compared with 60 weeks for patients with fewer mutation clusters (HR 2.9, 95% CI 1.2–6.4; *p* = 0.02). Moreover, the multivariate analysis further confirmed that high heterogeneity in terms of mutations was a prognosticator of poor PFS [58]. These data suggest that baseline tumor heterogeneity evaluated by ctDNA or ^18^F-FDG PET/CT could be both used as potential biomarkers of response to pyrotinib in HER2-positive MBC. PET imaging could provide a whole picture of metastatic disease and is a widely accepted diagnostic tool in BC while ctDNA analyses could reflect tumor status more dynamically. There were 13 patients in our study who had received both ctDNA NGS test and PET/CT before the treatment of pyrotinib. 10 of them showed abnormal gene variations. 90% of patients with high HI-inter disease on PET/CT had abnormal NGS results compared with 1/3 of patients in the low HI-inter group. Possible mechanisms for tumor temporal and spatial heterogeneity included differentiation state of cell-of-origin, cell plasticity, genetic evolution of cancer and microenvironment. Gene sequencing analyses offer an important way for a deeper understanding of the nature of tumor heterogeneity.

The SUVmax has been proven to be prognostic in various primary tumors [25,59,60,61,62]. Previous studies have shown that the SUVmax has predictive and prognostic value in patients with MBC [57,63,64]. Our study showed that SUVmax and TLG could also serve as potential markers for predicting pyrotinib treatment outcomes in patients with HER2-positive BC.

To the best of our knowledge, our study is the first to evaluate pyrotinib PFS in metastatic breast cancer by temporal HER2 concordance. Our results showed that patients with homogenous HER2 positivity had significantly longer PFS, followed by patients with gained HER2 positivity. Patients with HER2 negative conversion had little benefit from pyrotinib. Clinicians should pay more attention to the changes in a tumor’s biological behaviors during a patient’s therapeutic journal, which has profound implications for treatment outcomes. ^18^F-FDG uptake heterogeneity was applied to reflect tumor spatial heterogeneity. In our previous work, we have established novel parameters to represent the intra- and intertumoral heterogeneities among metastatic lesions on PET scans, and they have proven to be effective predictive markers in clinical practice. This study is the first to apply this method in HER2-positive BC. As far as we are concerned, this is also the first research to investigate the predictive value of ^18^F-FDG heterogeneity in patients with HER2-positive MBC. Heterogeneity in pretreatment PET/CT could help oncologists gain a better understanding of patients’ tumor heterogeneity and identify patients that would benefit from pyrotinib such that they could adapt treatment strategies for individual patients.

There are some limitations to this study. First, this study was an exploratory study based on a small cohort. Furthermore, the temporal heterogeneity in terms of IHC was only performed in patients with matched IHC results, which was 71% of the cohort. Validation is needed for further investigation. In addition, not all primary samples had a central pathology review of IHC results. Differences in the interpretation of IHC might introduce bias. Small changes in HR/HER2 expression were not considered as conversion in order to minimize the effect of technical reasons. In addition, enrollment criteria in this study included whole-body PET/CT scan prior to pyrotinib treatment. There may have been selection bias since PET/CT scans are more likely to be recommended in patients with a more complicated disease. Due to drug availability, only a small percentage of patients had prior pertuzumab or T-DM1 exposure in this population, which was consistent with the case in real-world practice in China. However, results from this study were difficult to be extrapolated directly to MBC patients in other areas. Last, HER2 heterogeneity at the gene level could provide more information. Translational studies investigating the biological mechanisms of inter- and intratumoral heterogeneity in HER2-positive MBC are still needed.

## 5. Conclusions

This article evaluated tumor heterogeneity in clinically applicable methods and investigated their impact on the efficacy of pyrotinib in MBC patients. Temporal tumor heterogeneity was evaluated by the discordance between primary and metastatic IHC results. Conversion of HER2 status was seen in 25% of these patients. Patients with homogenous HER2 positivity had significantly longer PFS, followed by patients with gained HER2 positivity. HER2 negative conversion, however, was predictive of poor outcome.^18^F-FDG uptake heterogeneity was applied to reflect spatial tumor heterogeneity among metastases. Baseline HI-intra and HI-inter could both predict the treatment efficacy of pyrotinib in patients with HER2-positive BC. This study underlines the importance of re-biopsy and adapting treatment with tumor heterogeneity taken into consideration.

## Figures and Tables

**Table 1:** Patients and tumor characteristics.

Characteristics	Patients (*n* = 51)	
	No.	%
**Age** (**years**)		
Median	54	
Range	23–74	
**Menopausal status**		
Postmenopausal	36	70.6
Premenopausal	15	29.4
**HR status ^a^**		
Positive	20	39.2
Negative	31	60.8
**De novo breast cancer**		
Yes	6	11.8
No	45	88.2
**Histological Grade ^b^**		
Grade 2	13	25.5
Grade 3	35	68.6
**Disease-free interval**		
<24 months	31	68.9
>24 months	14	31.1
**Number of metastatic sites**		
1	16	31.4
2	14	27.5
≥3	21	41.2
**Metastatic sites**		
Lung	10	19.6
Liver	12	23.5
Bone	23	45.1
Brain	10	19.6
**Visceral disease**	25	49.0
**Treatment line ^c^**		
1	21	41.2
2	23	45.1
≥3	7	13.7
**Previous anti-HER2** **treatment**		
Trastuzumab	47	92.2
Pertuzumab	13	25.5
Lapatinib	4	7.8
Trastuzumab emtansine	1	2.0

^a^ In patients with discordant HR status, the most recent results were presented. ^b^ Nottingham System, WHO 2019. Three patients did not have results. ^c^ Treatment line in which pyrotinib was administered. Abbreviations: HR, hormone receptor; No., Number.

**Table 2:** Analysis of factors associated with PFS.

Factors		No. of Patients	PFS (Months)	95% CI	*p*-Value
Clinical risk factors					
**Age**	≥54 years	26	13.4	7.2–19.5	0.456
	<54 years	25	14.4	9.1–19.7	
**HR status ^a^**	Positive	20	13.7	8.9–18.5	0.930
	Negative	31	13.4	8.8–17.9	
**Histological Grade**	Grade 2	13	10.6	9.7–11.5	0.365
	Grade 3	35	13.7	10.8–16.6	
**Disease-free interval**	>24 months	14	13.7	1.5–26.0	0.872
	<24 months	31	12.4	9.3–15.6	
**Treatment line ^b^**	First- or second-line	44	15.7	4.1–27.3	0.017 *
	Third- or later-line	7	10.6	10.2–11.1	
**Resistance to previous trastuzumab ^c^**	Yes	26	12.4	3.6–21.2	0.432
	No	25	15.7	9.4–22.06	
**No. of metastatic sites**	1	16	25.3	NR	0.015 *
	≥2	35	11.2	8.9–13.5	
**Visceral disease**	Yes	25	11.2	9.6–12.9	0.280
	No	26	16.8	7.4–26.2	
**Combinational agent**	Capecitabine	40	13.4	7.1–19.6	0.911
	Others	11	14.4	8.7–20.1	
**Tumor heterogeneity**					
**Temporal tumor heterogeneity between primary and metastatic disease**					
**HR status**	heterogeneous	10	11.1	8.6–13.6	0.887
	homogeneous	26	16.8	8.5–25.1	
**HR expression**	heterogeneous	15	13.7	9.5–18.0	0.541
	homogeneous	21	16.8	8.5–25.1	
**HER2 status**	heterogeneous	9	5.8	3.0–8.6	0.001 *
	homogeneous	27	16.8	4.5–29.1	
**HER2 expression**	heterogeneous	12	5.8	3.7–8.0	0.001 *
	homogeneous	24	NR	NR	
**Spatial tumor heterogeneity in terms of ^18^F-FDG uptake**					
**HI-intra**	>1.69	26	10.6	9.5–11.7	0.023 *
	<1.69	25	25.3	5.9–44.8	
**HI-inter**	>1.15	31	11.2	7.3–15.1	0.040 *
	<1.15	20	25.3	NR	

^a^ In patients with discordant HR status, the most recent results were presented. ^b^ Treatment line in which pyrotinib was administered. ^c^ Resistance to trastuzumab was defined as relapse during or within 6 months after adjuvant trastuzumab or progression within 3 months of trastuzumab treatment for metastatic disease [8]. Abbreviations: No., Number; PFS, progression-free survival; CI, confidence interval; NR, not reached; HR, hormone receptor; HER2, human epidermal growth factor receptor 2; FDG, fluorodeoxyglucose; HI-intra, intratumoral heterogeneity index; HI-inter, intertumoral heterogeneity index. * *p* < 0.05 is considered significant.

## Data Availability

The datasets generated and/or analyzed during the current study are not publicly available due to hospital policy but are available from the corresponding author on reasonable request.

## Supplementary Materials

The following supporting information can be downloaded at: https://www.mdpi.com/article/10.3390/cancers14163973/s1, Table S1: Associations between clinical factors and tumor heterogeneity; Table S2: Associations between PET parameters and PFS; Table S3: Association between temporal and spatial heterogeneity in terms of IHC results and ^18^F-FDG uptake.
