# Supplementary material for: Temporal Heterogeneity of HER2 Expression and Spatial Heterogeneity of 18F-FDG Uptake Predicts Treatment Outcome of Pyrotinib in Patients with HER2-Positive Metastatic Breast Cancer
Source: Cancers (Basel). 2022 Aug 17;14(16):3973. doi: 10.3390/cancers14163973 (PMC9406192; doi:10.3390/cancers14163973)
Supplement: Supplementary file 1 [file cancers-14-03973-s001.zip › cancers-1821404-supplementary.pdf]

Table S1. Associations between clinical factors and tumor heterogeneity.

Characteristics	Temporal tumor heterogeneity					Spatial tumor heterogeneity									
	Patients with heterogeneous HER2 status N=9		Patients with homogeneous HER2 status N=27		P value[a]	Patients with high HI-intra N=26		Patients with low HI-intra N=25		P value	Patients with high HI-inter N=31		Patients with low HI-inter N=20		P value
	No.	%	No.	%		No.	%	No.	%		No.	%	No.	%	
**Age**					1.000					0.328					0.654
≥54 years	5	55.6	13	48.1		15	57.7	11	44.0		15	48.4	11	55.0	
<54 years	4	44.4	14	51.9		11	42.3	14	56.0		16	51.6	9	45.0	
**Histological Grade**					1.000					0.202					0.784
Grade 2	3	33.3	8	32.0		9	34.6	4	18.2		8	28.6	5	25.0	
Grade 3	6	66.7	17	68.0		17	65.4	18	81.8		20	71.4	15	75.0	
**Disease-free interval[b]**					0.035*					0.731					0.213
<24 months	3	33.3	18	78.3		16	66.7	15	71.4		16	61.5	15	78.9	
>24 months	6	66.7	5	21.7		8	33.3	6	28.6		10	38.5	4	21.1	
**No. of metastatic sites**					0.392					0.057					0.003*
1	1	11.1	9	33.3		5	19.2	11	44.0		5	16.1	11	55.0	
≥2	8	88.9	18	66.7		21	80.8	14	56.0		26	83.9	9	45.0	
**Visceral disease**					0.706					0.886					0.029*
Yes	5	55.6	12	44.4		13	50.0	12	48.0		19	61.3	6	30.0	
No	4	44.4	15	44.6		13	50.0	13	52.0		12	38.7	14	70.0	
**Treatment line[c]**					1.000					1.000					1.000
1-2	8	88.9	22	81.5		22	84.6	22	88.0		24	77.4	20	100	
≥3	1	11.1	5	18.5		4	15.4	3	12.0		7	22.6	0	0	
**Resistance to previous trastuzumab[d]**					1.000					0.206					0.301
Yes	5	55.6	14	51.9		11	42.3	15	60.0		14	45.2	12	60.0	
No	4	44.4	13	48.1		15	57.7	10	40.0		17	54.8	8	40.0	

[a.] Chi-squared test or the Fisher exact test was applied. *p<0.05 is considered significant.

[b]In patients who received radical surgery. [c]Treatment line in which pyrotinib was administered. [ds]Resistance to trastuzumab was defined as relapse during or within 6 months after adjuvant trastuzumab or progression within 3 months of trastuzumab treatment for metastatic disease.

Table S2. Associations between PET parameters and PFS

Factors		No. of patients	PFS (months)	95% CI	p value
**SUVmax**	>7.96	38	11.1	6.6-15.6	0.008*
	<7.96	13	NR		
**SUVmean**	>3.43	43	11.2	7.4-15.0	0.077
	<3.43	7	NR		
**COV**	>0.15	27	11.1	7.2-15.0	0.026*
	<0.15	24	25.3	5.0-45.6	
**MTV**	>3.34 mL	26	14.4	10.9-17.9	0.865
	<3.34 mL	25	13.7	8.7-18.8	
**TLG**	>74.89 g	26	11.2	8.9-13.5	0.024*
	<74.89 g	25	NR	NR	

Abbreviations: PET, positron emission tomography; PFS, progression-free survival; No., Number; CI, confidence interval; SUVmax, maximum standardized uptake value; NR, Not reached; SUVmean, mean standardized uptake value; COV, coefficient of variance; MTV, metabolic tumor volume; TLG, total lesion glycolysis. * p< 0.05

Table S3. Association between temporal and spatial heterogeneity in terms of IHC results and ^{18}F-FDG uptake.

Factors		HI-inter Median (P25, P75)	Z P value	HI-intra Median (P25, P75)	Z P value
**HER2 status**	heterogeneous	3.44(2.27, 4.16)	-2.289	1.69(1.67, 1.76)	-0.785
	homogeneous	1.47(1.0, 2.61)	0.022*	1.69(1.63, 1.72)	0.432
**HR status**	heterogeneous	1.55(1.06, 3.36)	-0.071	1.69(1.65, 1.74)	-0.318
	homogeneous	1.94(1.0, 3.58)	0.943	1.69(1.65, 1.72)	0.751

Factors		HER2 status		P value[a]	HR status		P value
		heterogeneous	homogeneous		heterogeneous	homogeneous	
**HI-inter**	High	36.4%	63.6%	0.062	27.3%	72.7%	1.000
	Low	7.1%	92.9%		28.6$	71.4%	
**HI-intra**	High	25.0%	75.0%	1.000	31.3%	68.8%	0.481
	Low	25.0%	75.0%		25.0%	75.0%	

[a]Fisher's Exact Test was applied. Abbreviations: IHC, immunohistochemistry; FDG, fluorodeoxyglucose; HI-intra, intratumoral heterogeneity index; HI-inter, intertumoral heterogeneity index; HR, hormone receptor; HER2, human epidermal growth factor receptor 2. *p<0.05 is considered significant.
